# Approximate Atomic Green Functions

**DOI:** 10.3390/molecules26092660

**Published:** 2021-05-01

**Authors:** Stephan Fritzsche, Andrey Surzhykov

**Affiliations:** 1 Helmholtz-Institut Jena, Fröbelstieg 3, D-07743 Jena, Germany; 2 GSI Helmholtzzentrum für Schwerionenforschung, D-64291 Darmstadt, Germany; 3 Theoretisch-Physikalisches Institut, Friedrich-Schiller-Universität Jena, D-07743 Jena, Germany; 4 Fundamentale Physik für Metrologie, Physikalisch-Technische Bundesanstalt, D-38116 Braunschweig, Germany; andrey.surzhykov@ptb.de; 5 Institut für Mathematische Physik, Technische Universität Braunschweig, D-38106 Braunschweig, Germany

**Keywords:** atom, atomic cascade, atomic Green function, atomic structure, excitation scheme, ion, multi-photon, relativistic, second-order

## Abstract

In atomic and many-particle physics, Green functions often occur as *propagators* to formally represent the (integration over the) complete spectrum of the underlying Hamiltonian. However, while these functions are very crucial to describing many second- and higher-order perturbation processes, they have hardly been considered and classified for complex atoms. Here, we show how relativistic (many-electron) Green functions can be approximated and systematically improved for few- and many-electron atoms and ions. The representation of these functions is based on *classes* of virtual excitations, or so-called *excitation schemes*, with regard to given bound-state reference configurations, and by applying a multi-configuration Dirac-Hartree-Fock expansion of all atomic states involved. A first implementation of these approximate Green functions has been realized in the framework of Jac, the Jena Atomic Calculator, and will facilitate the study of various multi-photon and/or multiple electron (emission) processes.

## 1. Introduction

Various non-linear (second- as well as higher-order perturbation) processes have been observed during the past years but could often not be calculated in good detail for many ions, atoms or molecules of interest. Well-known second-order processes of this sort include, for instance, the multi-photon absorption and emission [1,2,3], the resonant [4] and two-photon ionization [5,6], the radiative and double Auger emission of atoms [7,8] and molecules [9], their (single-photon) double ionization [10,11,12] or the Rayleigh and Raman scattering of light [13,14,15], to name just a few. Until the present, however, most of these processes are not yet (well) understood *quantitatively* since, in perturbation theory, each additional order (beyond the first-order) typically requires an implicit summation (integration) over the full spectrum of the system. For complex atoms, and even more for molecules, this summation can be performed only approximately—and rather crude approximations, such as the independent-particle model or the restricted summation over just a few intermediate levels, were often made in the literature right from the very beginning.

As in first-order perturbation theory, most observables of such (non-linear) second- and higher-order processes are typically *expressed* in terms of so-called transition or scattering amplitudes that connect an initial bound state $|i\rangle$ of the atom or molecules with some final state $|f\rangle$. For the—elastic or inelastic—scattering of light on atoms or ions, for example, these transition amplitudes take the slightly simplified form [16]

(1) $$\begin{array}{ccc} M & = & \sum_{\nu }\;\frac{\langle f\left|\;O^{\left(\mathrm{emission}\right)}\;\right|\nu \rangle \;\langle \nu \left|\;O^{\left(\mathrm{absorption}\right)}\;\right|i\rangle }{E_{i}\;+\;\hbar \;\omega_{i}\;-\;E_{\nu }}\;, \end{array}$$

and which can be readily interpreted—the absorption of *one* photon first brings the atom into an intermediate state $|\nu \rangle$, and from which a *second* photon is subsequently re-emitted. Here, O denotes the electron-photon interaction (operator), $\;E_{i}\;$ and $\;E_{\nu }\;$ the total energies of the initial and intermediate states, and $\;\hbar \;\omega_{i}\;$ refers to the energy of the incident photon [17]. Moreover, the absorption and re-emission of the photons (may) need to be interchanged quantum-mechanically due to the complex-conjugated part of the interaction operators, leading to so-called crossed-term diagrams in many-body perturbation theory, and often only selected multipole components of the radiation field are considered. In practice, however, the summation (integration) remains the most challenging part of all numerical computations owing to large number of terms, while the (formal) need of *free-free* matrix elements in the construction of the intermediate states may hamper such an approach even further. It is *this* summation that can be *captured* by means of an approximate Green function and that lies in the focus of the present work. In addition, amplitudes quite similar to expression (1) also occur with $\;|f\rangle \;\equiv \;|i\rangle \;$ for a number of other atomic and molecular properties, such as the static and dynamic polarizabilities of an atom [18], the electric and magnetic susceptibilities [19], dispersion coeffients [20], shielding constants of nuclear moments, and at various places elsewhere. For molecules, furthermore, atomic continuum functions have been found relevant for estimating molecular Auger rates [21], angle-differential cross sections [22], or for testing ionization continuum models [23].

Both the frequent application of atomic Green functions and their inherent complexity make the intelligible access to and use of these functions very desirable. Of course, such a direct handling of the many-electron Green functions requires, first of all, their decomposition into building blocks that are suitable for atomic structure theory. Here, we shall describe how such approximate (relativistic) Green functions can be formulated within a symmetry-adopted basis of configuration state functions (CSF), and provided for practical computations. These approximate Green functions have been implemented moreover within the framework of the Jena Atomic Calculator (Jac), an open-source Julia package for doing relativistic atomic computations. This Jac toolbox [24,25] is based on the multi-configuration Dirac-Hartree-Fock (MCDHF) method [26], and it was designed right from the beginning for dealing with quite different requests in atomic (and partly molecular) theory.

In this work, the representation of approximate atomic Green functions is based on *classes* of virtual excitations with regard to given bound-state reference configurations, and by making use of a MCDHF expansion of all atomic states involved. Below, we shall explain how the—formally-defined—many-electron Green function can be decomposed quite readily into parts with well-defined symmetry and completeness properties. After a short theoretical account of a few selected properties of Green functions in Section 2, and especially on useful excitation schemes for atoms and ions with complex shell structures, emphasis is placed in Section 3 upon the representation and implementation of these functions in terms of proper data structures. These data structures are designed in order to support the application of the approximate (Green) functions for different atomic processes and for most, if not all, atoms or ions across the periodic table. As an example, we also show the (generation of an) approximate Green function for the two-photon excitation and ionization of atomic neon, along with a short discussion of its further application. Finally, a brief summary and conclusions are given in Section 4. Atomic units are used throughout in this work if not stated otherwise.

## 2. Theoretical Background

### 2.1. Dirac’s One-Electron Spectrum

For hydrogenic ions, the Coulomb-Green function has been frequently applied in the literature, both in the non-relativistic and relativistic atomic theory. Several representations are known especially for the *radial* part of these functions within position space [27,28]. For the Dirac Hamiltonian $\;H^{\left(\mathrm{Dirac}\right)}\;=\;c\;\left(\alpha \cdot p\right)\;+\;\beta \;c^{2}\;+\;V\left(r\right)\;$ and the Coulomb potential V(r)=−Z/r, the Coulomb-Green function is given by a 4×4-matrix, which satisfies the equation

$$\begin{array}{ccc} \left(H^{\left(\mathrm{Dirac}\right)}\left(r\right)\;-\;\varepsilon \;-\;c^{\;2}\;\right)\;G_{\varepsilon }\left(r,r^{\prime }\right) & = & \delta \left(r-r^{\prime }\right)\;I_{\;4}\;. \end{array}$$

Here, α and β are the well-known Dirac matrices, $\;I_{\;4}\;$ the 4×4 unit-matrix, and where—as usual in atomic structure theory—the rest energy $m\;c^{\;2}$ is not incorporated into the (total) electronic energy ε of the hydrogenic atom or ion. Solutions to this equation are often discussed in the literature in terms of the Whittaker or the Kummer functions of the first and second kind, but they can also be formally expressed by means of their *spectral* decomposition

(2) $$\begin{array}{ccc} G_{\varepsilon }\left(x,x^{\prime }\right) & = & \sum_{\begin{array}{c} \nu \end{array}}\;\int \;\frac{\psi_{\nu }\;\left(x\right)\;\psi_{\nu }^{+}\;\left(x^{\prime }\right)}{\varepsilon_{\nu }\;-\;\varepsilon }\;=\;\sum_{\begin{array}{c} n_{\nu }\left[\epsilon_{\nu }\right]\kappa_{\nu }m_{\nu } \end{array}}\;\int \;\frac{\langle x|n_{\nu }\kappa_{\nu }m_{\nu }\rangle \;\langle n_{\nu }\kappa_{\nu }m_{\nu }|x^{\prime }\rangle }{\varepsilon_{\nu }\;-\;\varepsilon }\;, \end{array}$$

and where x=(r,σ) denote the spatial and spin coordinates of the electron, while $\;n_{\nu }\left[\epsilon_{\nu }\right]\;$ indicates the summation over the discrete and the integration over the contineous part of the spectrum. Often, moreover, a radial-angular representation of the atomic spectrum with quantum numbers $\;\nu \;\equiv \;(n_{\nu }\kappa_{\nu }m_{\nu })\;$ is utilized, and as displayed on the right-hand side of Equation (2). Whereas the wave functions of the Dirac Hamiltonian are readily implemented for all energies, for example, the bound and free-electron states [29], the Green functions are often provided only for bound-state energies (ε<0) [30], even though a representation of the radial Green function in an analytical Sturmian basis has been worked out as well and can be applied quite readily [31].

As seen from the radial-angular (spectral) decomposition (2), already a single-electron Green function generally implies three *infinities* owing to the spatial degrees of freedom of the electron. In practice, therefore, any summation over the quantum numbers $\;(n_{\nu }\kappa_{\nu }m_{\nu })\;$ needs to be truncated at some principal quantum number $\;n^{\left(\max\right)}\;$ as well as to some proper list of angular momentum quantum numbers ${\;|\kappa |}^{\left(\max\right)}\;$. Often, this truncation is done implicitly by choosing a finite, for instance B-spline, basis for the numerical solution of the one-electron Dirac equation and, hence, a pseudo-representation of the full spectrum. For *N*-electron atoms and ions, in contrast, the 3N infinities of the associated Green functions are indeed a (very) serious challenge, and any truncation of these infinities must be based on good physical *insight* into the particular application as well as into other approximations that need to be done in order to keep computations feasible.

### 2.2. Approximate Many-Electron Green Functions

Formally, of course, the Green function calculus is known as a very powerful and extensive mathematical machinery for solving inhomogeneous boundary-value problems. However, any detailed discussion of this machinery is well beyond the scope of this work. Instead, here we shall take a *pragmatic* viewpoint, namely, that any properly truncated spectral decomposition

(3) $$\begin{array}{ccc} G_{E}\left(x_{1},x_{2}\ldots ;\;x_{1}^{\prime },x_{2}^{\prime },\ldots \right) & \equiv & G_{E}\left(X;\;X^{\prime }\right)\;=\;\sum_{\begin{array}{c} \nu \end{array}}\;\int \;\frac{\langle X|\alpha_{\nu }\;J_{\nu }M_{\nu }\rangle \;\langle \alpha_{\nu }\;J_{\nu }M_{\nu }|X^{\prime }\rangle }{E_{\nu }\;-\;E}, \end{array}$$

based on relativistic and approximate, many-electron atomic state functions (ASF) $\;\langle X|\alpha_{\nu }J_{\nu }M_{\nu }\rangle \;$ in position space $\;X\;=\;x_{1},x_{2},\ldots \;$, also represents a (relativistic) *approximate* Green function of an atom or ion. Here, $\;J\;\equiv \;J^{P}\;$ is a short-hand notation of the total angular momentum *J* *and* the parity *P* of the many-electron state, while *M* refers to the projection of the angular momentum, and α to all other quantum numbers that are needed in order to determine the states uniquely. This compact notation resembles expression (2) but enables us to readily include the coupling (fine-structure) of the electrons and to enlarge the many-electron Green functions in a systematic manner without any change in the underlying classification of the atomic levels. In practice, of course, this notation just “moves” the inherent complexity of all many-electron Green functions and the evaluation of transition amplitudes into the construction of the symmetry-adopted CSF bases [26,32], their diagonalization [33,34] as well as the computation of the (so-called) angular coefficients [35]. Apart from the truncation of the 3N quantum numbers in summation (3), therefore, the term *approximate* also refers to the representation of the ASF $\;\langle X|\alpha_{\nu }J_{\nu }M_{\nu }\rangle \;$ as well as to the detailed pole structure (in the complex plane) in order to account for (additional) boundary conditions. Here, we need not to discuss these issues in much further detail as they are central to all (relativistic) atomic structure codes.

With this pragmatic view in mind, any well truncated summation (3) will be seen here as an approximate many-electron Green function as long as the asymptotic behaviour (of the electron waves with positive energy), the symmetry and *completeness* of the ASF in this expansion can be properly explained. While the one-electron orbitals are readily generated for any (self-consistent) central-field potential, the symmetry of the atomic states $|\alpha_{\nu }\;J_{\nu }M_{\nu }\rangle$ arises, as usual, from the diagonalization of a properly chosen Hamiltonian matrix, and whose set-up and computation may significantly be simplified by using a symmetry-adopted basis of CSF. This approach has been realized especially in the MCDHF method for several decades [36,37], and is employed also within the Jac program below [24,25]. The “completeness” of the (various spectra of) ASF is of course elusive but can be explained quite similar as for any restricted or complete active-space method [38,39]. Finally, the asymptotic behaviour of the atomic states for $\;r_{N}\to \infty \;$ is mainly relevant for the (auto-) ionization [40,41] and electron capture processes [42], and especially if angle- and polarization-resolved properties are considered for the free electron [43,44]. Further work will need to be done in order to understand this asymptotic behaviour in good detail.

**Figure 1:**
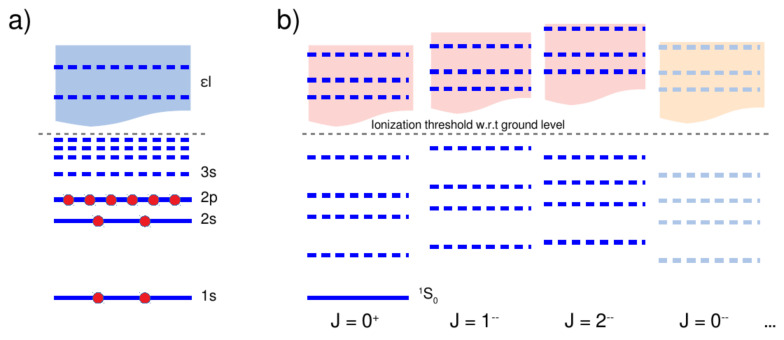
Comparison of the single- and many-electron spectra (continua) of atoms and ions with complex shell structure. In the relativistic theory, the (**a**) single-electron energies (solid and dashed blue lines) follows from the solution of the Dirac equation in a proper central-field potential V(r), and which are designated in the (standard) $\;1s,\;2s,\;2p_{1/2,\;3/2},\;\ldots \;$ subshell notation, analogue to the hydrogenic atom. Above of the ionization threshold, ε=0, there is a single-electron continuum (light blue box) for each symmetry (κ,m), and which is formally often captured by means of a finite summation (integration) over the pseudo-states. (**b**) For many-electron atoms, quite similar, the bound-state solutions (solid and dashed blue lines) are still diagonal in the total symmetry $\;J\;\equiv \;J^{P}\;$ of the atomic state functions but now requires a proper coupling (and construction) of the CSF; see text for further details. Again, a many-electron continuum (light red boxes) is associated with each symmetry (J,M), although at slightly different threshold energies with regard to the ground-state level. While the subshell occupation (red circles in the left panel) of the many-electron states just refer to the single-electron spectrum, only their total symmetry (and energetic order) are relevant for the classification and distinction of the many-electron continua. The selected occupation and symmetries in both panels refer to neon-like ions with a $\;1s^{2}2s^{2}2p^{6}\;{\;}^{1}S_{0}\;$ ground level. For the $\;1s^{2}2s^{2}2p^{6}\;{\;}^{1}S_{0}\;+\;2\;\hbar \;\omega \;\to \;1s2s^{2}2p^{6}3d\;{\;}^{3}D_{1,2,3}\;$ two-photon absorption of these ions, the dominant E1E1 transition amplitude (1) is based on the $\;J^{P}\;=\;1^{-}\;$ symmetry channel, while the $\;2^{-}\;$ channel need to be taken into account, in addition, if the E1M2 multipole excitations are considered as well. Other channels (continua) are not relevant for the example from Section 3.2.

The rotational symmetry and parity of the ASF $|\alpha_{\nu }\;J_{\nu }M_{\nu }\rangle$ split each approximate Green function into separate channels (continua) of well-defined symmetry $\;J\;\equiv \;J^{P}\;$, and as shown in Figure 1b. This figure compares the single- and many-electron spectra (continua) of atoms and ions with complex shell structure and briefly explains the role of the (total) symmetry of the states. Analogue to the one-electron Coulomb-Green function (2), an infinite number of channels generally occur for every atom or ion, and including channels of both parities, P=±1. In practice, however, only a few of these channels (continua) are usually relevant for any non-linear interaction process, either because of the symmetry of the underlying interaction operators or due to further limitations in the theoretical description. For the two-photon excitation and ionization of a 1s electron from neon-like ions [5,45], for example, Figure 1 displays the $\;J^{P}\;=\;1^{-}\;$ symmetry channel due to a E1E1 excitation as well as the $\;2^{-}\;$ channel if, in addition, one wishes to account for E1M2 multipole excitations.

While the decomposition of the atomic Green function into symmetry blocks (channels) is quite straightforward to *do*, the explicit representation of the ASF $\langle X|\alpha_{\nu }\;J_{\nu }M_{\nu }\rangle$ in expression (3) requires much further care. For this representation, the proper couplings of *all* electrons need to be considered in the set-up and computation of this Hamiltonian matrix. This is particularly true if some atomic process leads to a multiple ionization (or capture) of electrons, such as the double Auger emission. The distinction of different *approaches* in the construction of the Hamiltonian matrix will be further discussed below. Here, let us just resume that any symmetry channel (continuum) of the Green function is practically equivalent to the set of atomic eigenvectors $\;\{E_{\nu },\;|\alpha_{\nu }\;JM\rangle \}\;$ with fixed total symmetry (J,M), and which formally belongs to both, the bound-state spectrum of the atom $\;(E_{\nu }\;<\;0)\;$ as well as to its continuous part (scattering states; $\;E_{\nu }\;\geq \;0\;$), and with 1,2,... or, possibly, even more electrons within the continuum. All these levels are normally constructed from a single set of one-electron orbitals, and which need first of all to be chosen in such a way to represent the bound-state levels of the given configurations reasonably well. From these sets of atomic eigenvectors, the second and higher-order transition amplitudes can then be obtained by just evaluating the corresponding many-electron matrix elements, quite analogue as in first-order perturbation theory.

The many-electron Green function (3) is closely related also to the (potential) scattering of atoms

$$\begin{array}{ccc} H\;|\Psi^{+}\rangle & = & E\;\;|\Psi^{+}\rangle , \end{array}$$

based on some properly chosen many-electron Hamiltonian H, the collision energy *E* and by including suitable scattering boundary conditions as indicated by the + sign above. A formal solution to this Schrödinger equation is given by the well-known Lippman-Schwinger equation in terms of the initial state $|\Psi_{o}\rangle$ and the many-electron Green operator $\;G_{E}^{\;+}\;$ [46].

(4) $$\begin{array}{ccc} |\Psi^{+}\rangle & = & |\Psi_{o}\rangle \;+\;\frac{1}{E\;-\;H\;+\;i\eta }\;V\;|\Psi_{o}\rangle \;+\;\ldots \;\equiv \;|\Psi_{o}\rangle \;+\;G_{E}^{+}\;V\;|\Psi_{o}\rangle \;+\;\ldots \;, \end{array}$$

and where V describes the interaction (potential) as seen by the atom. Here, the small positive parameter η>0 ensures that the boundary conditions of an overall outgoing wave are taken into account. In single-electron excitation and (auto-) ionization processes, in contrast, these boundary conditions are usually treated less formally, although quite similar, in terms of the scattering phases of the incoming or outgoing electrons [40,47,48]. Alternatively, ansatz (4) can be combined also with an optical potential [49], and which (may) help avoid the cumbersome procedure of adjusting the boundary conditions for many-electron atoms.

### 2.3. Selection of Subspaces

Having an approximate Green function characterized by a (finite) number of symmetry channels, that is, properly constructed *sets* of many-electron ASF with well-defined symmetry J and energetic order, we just need to deal with those subspaces (of the many-electron Hilbert space) that have to be taken into account for a particular application. Obviously, the selection and construction of these subspaces requires some physical understanding of the underlying atomic process. For most processes, this construction can be made by specifying suitable *schemes* (classes) of virtual excitations or de-excitations with regard to some reference configurations (or states). For a careful and systematic construction, moreover, it is desirable to start from a *set* of reference configurations and to generate virtual excitations due to a few simple rules. Of course, these rules should distinguish also how and to which extent correlations among the bound and continuum electrons are taken into account within the particular Green function representation. Table 1 displays and briefly explains a few of these excitation schemes which help control the generation of approximate Green functions and which have been implemented in the Jac program below. These schemes are based on the atomic shell model and, especially, on the well-known concept of (non-relativistic) electron configurations. They are used to describe how the shell occupation can be modified and how many of the electrons may be released into the continuum. In other words, each approximate Green function can be classified also in terms of the (maximum) number of free electrons as well as a pre-specified *excitation scheme*. This scheme is then applied in order to generate all (non-relativistic) configurations that are considered in the associated many-electron (CSF) basis.

For example, the excitation of a *single* electron from a given set of (non-relativistic) configurations includes all those configurations with a replacement of *one* electron from an occupied subshell into another occupied or yet unoccupied shell. Although the number of electrons of the generated configurations is still the *same* as before, up to *one* free electron may now occur in the *N*-electron scattering states. Indeed, several of these excitation schemes have been implemented below in order to specify and generate suitable (lists of) CSF bases for the selected channels with total symmetry J. Apart from the construction of approximate Green functions, the same or quite similar excitation schemes are required also for the computation of atomic cascades [50,51,52], or the construction of restricted active spaces [53], in order to incorporate inter-electronic interactions in a balanced but user-controlled manner.

The use of excitation schemes classifies each Green function first of all in terms of electron configurations, that is, in terms of occupation numbers of subshells (nℓ), such as the $\;1s^{2}2s^{2}2p^{6}\;$ ground configuration of neon-like ions. Of course, further restrictions can still be placed upon the principle and orbital angular momentum quantum numbers, and they are often needed in order to keep the size of the CSF basis feasible. These restrictions have to be specified separately. Below, we shall typically fix a maximum principle quantum number $n^{(}\max)$, up to which subshells can be occupied, although the associated (pseudo-) orbitals might belong also to the continuum, if a finite one-electron basis is applied. In contrast, the orbital angular momenta $\;\ell \;\in \;[\ell_{1},\;\ell_{2},\;\ldots ]\;$ need to be given explicitly to allow the subshells to be restricted to just one or a few partial waves. The detailed contribution of these one-electron orbitals (basis) to a particular Green function channel with total symmetry J follows however only from the coupling of the corresponding—now relativistic—subshells (nκ), and where the relativistic angular momentum quantum number κ=∓(j+1/2) for j=ℓ±1/2 already includes the spin degree of freedom. For reference configurations with several open shells, moreover, the number of the so-generated CSF becomes often quite large (say, >$10^{\;4}$) already for moderate $n^{(}\max)$, and even for each single symmetry J, and this rapid increase then limits the excitation schemes that remain practical for further computations.

For a given set of configurations, the number of CSF is of course geometrically fixed owing to the coupling (rules of the *equivalent* electrons) within each subshell as well as the coupling of the various subshell states. In fact, the construction of such CSF bases is a standard task in atomic structure theory. We here make use of symmetry-adopted CSF $|\alpha \;JM\rangle$ with well defined angular momentum and parity as implemented, for example, in the Grasp [36] and Ratip codes [54]. Apart from the geometrical construction of the CSF basis, however, the particular representation of the atomic states may still differ significantly from each other because of the use of various (approximate) Hamiltonians in the set-up of the energy matrices, the limitations of the one-particle basis or the way, how the matrix elements are computed with CSF that are embedded in the continuum. We shall discuss below a few suitable approaches for the computation of these ASF and, hence, the representation of the symmetry channels (continua) with fixed J, though further work is likely needed to better understand how different approximations will affect the use of these Green functions in different applications.

## 3. A First Implementation of Approximate Green Functions

### 3.1. Finding a Language for Many-Electron Atoms

The inherent complexity of all (many-electron) Green functions, outlined above, already suggests that a modern and efficient (computer-) framework is needed in order to deal with selected, or perhaps even most, of the atoms and ions across the periodic table. Today, Julia [55,56] is known as such a framework and programming language that was designed for bringing together (high) performance and productivity, and that enables the user to gradually learning fresh concepts in scientific computing [57]. Indeed, Julia includes a number of very powerful features that are common to so-called *productivity* languages, such as dynamic types, optional type annotations, type-specializing, just-in-time compilation of code, dynamic code loading as well as garbage collection.

The Jac toolbox [24,25] from above is entirely implemented in Julia and provides tools for performing atomic (structure) calculations of different kind and complexity. In its original design, we first of all aimed for developing a high-level language that (i) is simple enough for both, seldom as well as more frequent use of this toolbox, (ii) emphasizes the underlying physics, and (iii) avoids most technical slang that is often unnecessary but rather common to most electronic structure codes. These goals are all relevant also for the generation and application of approximate Green functions. Therefore, let us now describe how (instances of) approximate Green functions can be generated within Jac and subsequently applied in order to obtain the cross sections, rates or other properties of interest. In Section 2.2, we already saw that each Green function comprises different channels (continua) with well-defined total symmetry J, and which each refers to a set of atomic levels $\;\left\{E_{\nu }^{\left(J\right)},\;|\alpha_{\nu }^{\left(J\right)}M^{\left(J\right)}\rangle \right\},\;\nu \;=\;1,\ldots ,\nu^{\;(J)}\;$, all of the same total symmetry J. Using this notation, an approximate Green function is then given by an array (list) of *k* such channels and formally written as

(5) $$\begin{array}{ccc} & & \;\left[\left\{E_{\nu }^{\;(J_{1})},\;|\alpha_{\nu }^{\left(J_{1}\right)}M^{\left(J_{1}\right)}\rangle ,\;\nu =1,\ldots ,\nu^{\left(J_{1}\right)}\right\},\;\left\{E_{\nu }^{\;(J_{2})},\;|\alpha_{\nu }^{\left(J_{2}\right)}M^{\left(J_{2}\right)}\rangle ,\;\nu =1,\ldots ,\nu^{\left(J_{2}\right)}\right\},\;\ldots \right]\\ & \;⟺\; & G_{E}\;=\;\sum_{\begin{array}{c} \nu \end{array}}\;\int \;\frac{|\alpha_{\nu }\;J_{\nu }M_{\nu }\rangle \;\langle \alpha_{\nu }\;J_{\nu }M_{\nu }|}{E_{\nu }\;-\;E}\;\equiv \;\sum_{J}^{J_{k}}\;\sum_{M=-J}^{J}\;\sum_{\nu }^{\nu^{\;(J)}}\;\frac{|\alpha_{\nu }\;JM\rangle \;\langle \alpha_{\nu }\;JM|}{E_{\nu }^{\;(J)}\;-\;E}\;. \end{array}$$

In more detail, the *i*-th channel of an approximate Green function is represented by a list of many-electron (pseudo-) levels $\;\left\{E_{\nu }^{\;(J_{i})},\;|\alpha_{\nu }^{\left(J_{i}\right)}M^{\left(J_{i}\right)}\rangle ,\;\nu =1,...,\nu^{\;(J_{i})}\right\}\;$. Moreover, all channels are based on the same excitations, for example, the same set of (nonrelativistic) configurations, and have up to $\;n\;=\;n^{\left(\max\right)}\;$ electrons *within* the continuum. Obviously, such a list of many-electron levels is directly applicable also for subsequent numerical computations.

### 3.2. Data Structures for the Representation of Green Functions

Properly designed data structures are a key for defining useful objects (building blocks) for the computation and their straightforward data transfer within a program. For this reason also, Jac [24] is built upon a rather large number of well-designed data structures, which enable one to describe (and deal with) the electronic structure and processes of atoms and ions with complex shell structures. A few prominent examples are an Orbital to represent the quantum numbers and radial components of (one-electron) orbital functions, an (atomic) Basis to specify a whole set of CSF, or a Level for a full representation of a single ASF: $\;E,\;|\alpha \;JM\rangle \;$. Other data structures, such as an Atomic.Computation are employed to describe and control the calculation of atomic level structures, their properties as well as various processes among these levels. These data structures, already available in Jac, need to be extended here in order to support the generation and application of approximate Green functions.

**Figure 2:**
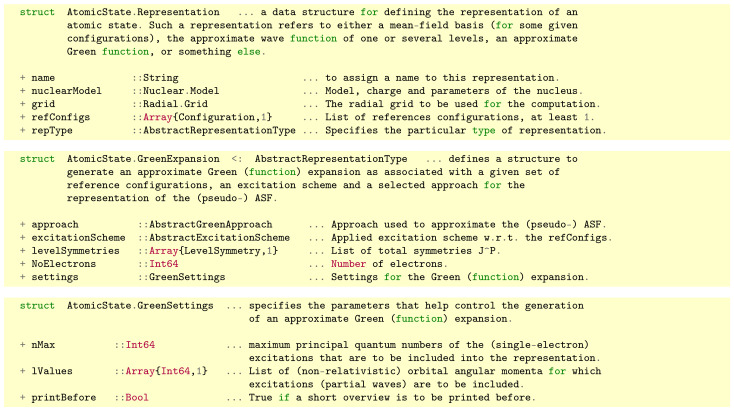
Definition of the data structures AtomicState.Representation (**upper panel**) to select and specify a representation of an atom or ions, based on a set of reference configurations. One particular representation is the AtomicState.GreenExpansion (**middle panel**) that specifies an approximate Green function in terms of an excitation scheme and a selected approach for the computation of the (pseudo-) ASF. Finally, the data structure AtomicState.GreenSettings (**lower panel**) enables the user to control the particular size of the approximate Green function.

In Jac, an approximate Green function is generated by means of a GreenExpansion, as shown in the middle panel of Figure 2. This (Green) expansion is a special type of an atomic Representation from the module AtomicState, cf. the upper panel of Figure 2. Here, a representation generally refers to the quantum-mechanical—and typically also numerical—formulation of either an atomic mean-field basis, a set of state functions or just an approximate Green function (within this work), and from which all the desired properties of an atom or ion can be derived. Apart from a name (string), such a numerical representation asks the user for the nuclear model, the (radial) grid for all computations, a list of reference configurations as well as the selected type, here a GreenExpansion <: AbstractRepresentationType. For this Green expansion, moreover, we then have to designate an approach::AbstractGreenApproach, a suitable excitationScheme::Abstract ExcitationScheme (with regard to the given reference configurations) as well as the list of total LevelSymmetry’s and the number of electrons within the system. The numerical generation of the Green functions can be further controlled by the settings::GreenSettings as shown in the lower panel of Figure 2. These settings enable the user to control the particular size and to truncate the number of ASF in each Green function channel by:

- a maximum principal quantum number $\;n^{\left(\max\right)}\;$ for all virtual orbitals, and which applies to all symmetry blocks κ from the single-electron spectrum;
- selecting a list of orbital angular momenta $\;[\ell_{1},\;\ell_{2},\;\ldots ]\;$ as considered for virtual excitations by the given (de-) excitation scheme.

Another truncation is made implicitly by the excitation scheme itself owing to its (maximum) number of *free* electrons. A few useful excitation schemes were shown in Table 1 and briefly explained in Section 2.3, though further schemes might be *added* in the future if they will facilitate new applications of the Jac toolbox. At present, we have restricted ourselves to Green function representations with either *no* or just *one* electron in the continuum, in line with the computation of the standard radial integrals in Jac. All subfields of the data structures in Figure 2 are specified quite analogue to the description in Section 2 and will facilitate the use of these functions in other applications.

Once an approximate Green function has been specified in terms of the Representation above, it can be readily computed just by typing [58]


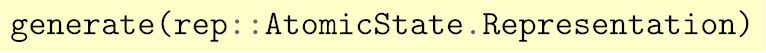


**Figure 3:**
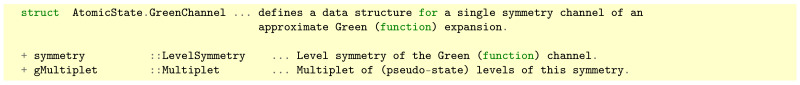
Definition of the data structure AtomicState.GreenChannel in Jac that help retain the representation of a single channel (continuum) of the approximate Green function (5).

This function call then returns a list of GreenChannel’s as displayed in Figure 3. As seen from this figure, each channel is internally represented as a Multiplet of (pseudo-state) ASF, all of which share the same symmetry::LevelSymmetry and all of which are directly applicable for numerical computations.

In practice, each of these channels typically includes a rather large number of pseudo-ASF (levels) in order to properly represent the (complete) continuum with total symmetry J. Indeed, the set-up and computation of these multiplets, including the full diagonalization of the associated Hamiltonian matrix, is computationally the most demanding part in the generation of approximate Green functions. Therefore, several *approaches* are distinguished within Jac in order to determine how the requested sets of many-electron levels $\;\left\{E_{\nu }^{\;(J_{i})},\;|\alpha_{\nu }^{\left(J_{i}\right)}M^{\left(J_{i}\right)}\rangle ,\;\nu =1,\ldots ,\nu^{\left(J_{i}\right)}\right\}\;$ with $\;J_{i}\;\in \;\left[J_{1},\;J_{2},\;\ldots \right]\;$ are to be generated and how much of the electron-electron interaction should to be taken into account in order to represent *both*, the bound and continuous part of the channel. Three such approaches are presently designed and (partly) supported by the Jac toolbox [cf. middle panel of Figure 2]. They make use of a:

(a) Diagonal CSF basis without any configuration interaction (SingleCSFwithoutCI):   This is a fast, though very rough, approximation, in which each CSF with total symmetry J just represents a single level $\;E_{\nu }^{\;(J_{i})},\;|\alpha_{\nu }^{\left(J_{i}\right)}\rangle \;$ of the spectrum. In this simple approximation, however, only the diagonal matrix elements of the Hamiltonian need to be computed.
(b) Basis that includes configuration interactions only between bound-state orbitals (CoreSpaceCI):   For each channel J, a full Hamiltonian matrix is diagonalized for all those CSF that are built only from bound-state orbitals, while just the diagonal matrix elements are estimated for computed for all other CSF with at least *one* free electron, ε>0.
(c) Full Hamiltonian, but where the electron-electron interaction is damped (DampedSpaceCI) in the radial coordinate *r* by a user-specified convergence factor $\;e^{-\tau \;r}\;$. This convergence factor applies to both, the bound and free-electron orbitals. For a proper choice of τ, however, this factor mainly affects the interaction with the continuum (and between pseudo-state which are embedded *within* the continuum). The use of such a *damping function* in the Slater integrals (and, possibly also in the Breit integrals—although this has not been realized so far) ensures that the bound-bound, bound-free and free-free interactions are formally treated on equal footings.

Further approaches for the (generation of the) Green function channels might be considered in the future if the need arises due to newly emerging applications.

### 3.3. Example: Two-Photon Excitation and Ionization of Atomic Neon

The two-photon absorption or decay of atoms are well-known second-order processes, although they are still slightly simpler than most other nonlinear processes, since they combine two bound states with no free electron, neither in the initial nor final state. The two-photon absorption, for instance, is described by a transition amplitude [1],

(6) $$\begin{array}{ccc} M^{\left(M_{2},M_{1}\right)}\left(\alpha_{i}J_{i}\to \alpha_{f}J_{f}\right) & = & \sum_{J}\;\sum_{\nu }\;\frac{\langle \alpha_{f}J_{f}∥\;O^{\left(M_{2},\;\mathrm{absorption}\right)}∥\alpha_{\nu }J\rangle \;\langle \alpha_{\nu }J∥\;O^{\left(M_{1},\;\mathrm{absorption}\right)}∥\alpha_{i}J_{i}\rangle }{E_{i}\;+\;\hbar \;\omega_{1}\;-\;E_{\nu }}\;, \end{array}$$

quite similar to Equation (1), and with a second term in which the photons are exchanged (1↔2). In this form of the transition amplitude, we now make the symmetry of the initial, intermediate and final levels more explicit, and also omit the magnetic quantum numbers (by using *reduced* matrix elements instead) as the associated summation can be treated algebraically by means of Wigner symbols and the Wigner-Eckart theorem. In the amplitude (6), moreover, the multipoles M∈{E1,M1,E2,…} are shown explicitly as they naturally arise from the standard decomposition of the radiation field in the electron-photon interaction. The summation (integration) over the intermediate states also shows the expansion into Green function channels of well-defined symmetry J, and for which the summation over $\nu \;=\;1,\;\ldots ,\;\nu^{\left(J\right)}$ has to be performed separately for each channel. It is this comprehensible re-writing of the (many-electron) transition amplitudes that makes a simple access to the (numerical) Green function channels so useful. From this or some similar form of the transition amplitudes, moreover, the symmetries J of the necessary Green function channels can be read-off easily. For example, the $\;1s^{2}2s^{2}2p^{6}\;{\;}^{1}S_{0}\;+\;2\;\hbar \;\omega \;\to \;1s\;2s^{2}2p^{6}3d\;{\;}^{3}D_{1,2,3}\;$ two-photon absorption of neon-like ions will be dominated by the $\;M^{\left(E1,\;E1\right)}{(}^{1}S_{0}\to {\;}^{3}D_{1,2,3})\;$ amplitude *via* the $\;J\;=\;1^{-}\;$ Green function channel, and which should include (at least) the $\;1s\to np_{\;1/2,\;3/2}\;$ excitations, in line with the excitation scheme DeExciteSingleElectron [cf. Table 1]. Apart from the bound-state (Rydberg) excitations 1s→3p,4p,…, this scheme should include a good representation of the $\;\varepsilon p_{\;1/2,\;3/2}\;$ one-particle (pseudo-) spectrum, and as readily obtained within a finite (B-spline) basis. If, in addition, one wishes to take into account also $\;M^{(E1,\;M2)\;}$ transition amplitudes, the $\;J\;=\;2^{-}\;$ Green function channel will be required as well, and which should then include the excitations into $\;1s\to nf_{\;5/2}\;$ as well.

In Jac, we can readily obtain these approximate (Green function) channels for neon-like ions by the following short Julia script:


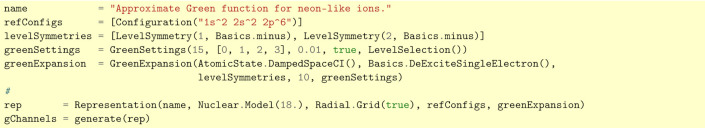


**Figure 4:**
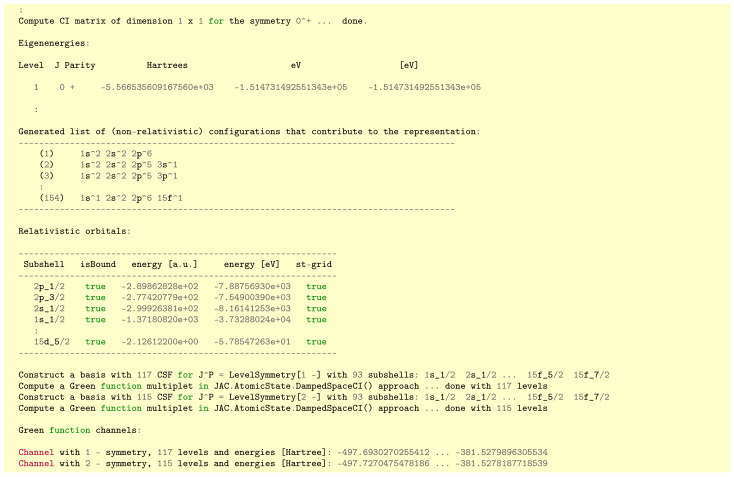
Selected printout from the example in Section 3.3.

As explained in Section 3.2, here, we just need to provide a name (string), the nuclear model, the (radial) grid parameters and the configurations as general input for all (atomic) Representation’s in Jac. For neon-like ions, we enter the $\;1s^{2}2s^{2}2p^{6}\;$ configuration and the Basics.DeExciteSingleElectron() scheme in order to include all excitations of a single electron into subshell (nℓ) with $\;n\;\leq \;n^{\left(\max\right)}\;=\;15\;$ and ℓ=0,1,2,3. This specification makes then use of all orbitals 1s,2s,2p,…,15s,15p,15d,15f into which virtual excitations are taken into account due to the replacement of a single electron, starting from $\;1s^{2}2s^{2}2p^{6}\;$. We also compute and diagonalize the full Hamiltonian for each channel, as designated by AtomicState.DampedSpaceCI(), but with a convergency-accelerating factor $\;e^{-0.01\;r}\;$. With this input, the Green function expansion is formally specified and can now be readily generated. Figure 4 shows parts of the output that is printed to screen (or, more precisely, the standard out stream). The program first computes a self-consistent field (SCF) for all levels from the reference configurations, here the $\;1s^{2}2s^{2}2p^{6}\;{\;}^{1}S_{0}\;$ ground level of neon-like Ar ${}^{8+}$ ions. It then generates (and displays) the list of non-relativistic configurations owing to the given excitation scheme. The SCF also determines the relativistic orbitals from the one-electron (pseudo-) spectra that are taken into account. The computationally most demanding part refers, however, to the set-up and diagonalization of the Hamiltonian matrix that, in turn, is carried out for each channel, and where the concrete number of levels arise from the coupling of the equivalent and non-equivalent electrons. Here, we restrict the CSF basis to quite a moderate size (≥100), although much larger bases could be obtained by increasing $\;n^{(}\max)\;$ or by allowing other one- and two-electron excitations. After the *generation* of all channels, the variable gChannels contains a list (array) of these Green function channels, and which each provide a multiplet that is suitable for a numerical summation (integration) over the associated many-electron levels as needed for the amplitude (6).

The *quality* of the generated Green functions can be checked first of all by means of the (total) energies of the atomic levels $\;(E_{\nu },\;|\alpha_{\nu }J_{\nu }M_{\nu }\rangle )\;$, that form the individual symmetry channels. These energies can be readily extracted from the final GreenChannel’s and compared with experiment (as far as available). Table 3 compares the excitation energies of the low-lying levels from the Green function channel $\;J=1^{-}\;$ with data from NIST [59]. These energies are compared for three computational models for generating this channel by using different approximations and settings. The use of a (I) diagonal CSF basis with $n^{\left(\max\right)}=15$ and ℓ=[0,1,2]; (II) the same but for the full Dirac-Coulomb Hamiltonian; and (III) the Dirac-Coulomb Hamiltonian, again, but by including additional excitations with regard to the $\;1s^{2}2s^{2}2p^{6}\;$ reference configuration. Model (III) already results in a rather large representation of the Green function channel $\;J=1^{-}\;$ with a total of 1532 ASF. Further relativistic corrections due to the Breit interaction or even quantum-electrodynamical estimates could be added to this representation, in principle, but will need further work to better understand their benefits for different applications of these approximated Green functions.

Our example above displays the generation of the $\;J\;=\;1^{-},\;2^{-}\;$ channels, although still in a rather crude approximation. A better representation of the continuum could be obtained by:

- Including a larger set of virtual excitations; here, the excitation scheme DeExciteTwoElectrons() also incorporates double excitations and, hence, further electronic correlations.
- An improved computation of the Hamiltonian matrix [33]; apart from a better account of the electron-electron interaction, this refers first of all to the treatment of the CSF, that are embedded into the continuum, and eventually also to the *negative* continuum, if one needs to go beyond the *no-pair* Hamiltonian.
- Enlarging the number of (pseudo-) orbitals in the representation of the one-electron spectra; this is done by increasing $\;n^{\left(\max\right)}\;$ for all channels or by a larger number of orbital angular momenta in the construction of the configuration lists. The user can affect the number and energetic position of the one-electron (pseudo-) orbitals by choosing proper grid parameters, although a direct preference of certain energy intervals is less obvious to achieve for these orbitals [60].
- Providing a better continuum for the channels; here concepts and the experience from (restricted) active-space computations might be helpful in the future.
- Supporting the hyperfine splitting or other symmetries owing to the additional occurrence of external fields already into the Hamiltonian, that is, into the representation of the corresponding (pseudo-) states.
- Adopting more advanced approaches, such as the *K*-matrix scattering theory [61], in order to make the Green functions applicable also to general scattering problems.

While all of these items indicate possible improvements in the representation (and usually also a sizeable increase in the number of ASF) for each channel, more often than not, they are unfeasible in practice. It generally requires physical insight and, perhaps, some prior experience of the user with the computation of the given process to properly select the input for the generation of approximate Green functions. Further work will likely be needed in order to better understand how the current limitations in the set-up of these functions can be *softened*, and how they can be made suitable for the accurate computation of nonlinear processes.

### 3.4. Use of Approximate Green Functions

Various (nonlinear) processes and related properties have been mentioned in the introduction above and formally require a single or multiple summation over a complete spectrum of the atom or ion of interest. Table 4 displays a few selected second-order properties and (atomic) processes whose transition amplitude request for the summation over different continua. They are briefly explained from a *physics* perspective in this table, while further details about their cross sections, rates, angular distributions, and so forth, still need to be worked out, and this applies especially for the evaluation of these transition amplitudes within the framework of the MCDHF method. Moreover, several of these processes involve *free* electrons, either in the initial and/or final state. Very little is known so far about the proper description of all processes, for which *two* or more electrons belong to the continuum. While the given classification of the (approximate) Green function channels might indeed help to deal with these processes also quantitatively, further work on the computation of the continuum-continuum matrix elements will be required and may perhaps even restrict the use of such summations.

## 4. Conclusions

In this work, we have shown how relativistic (many-electron) Green functions can be approximated and systematically enlarged to few- and many-electron atoms and ions. The representation of these approximate functions is based on different classes (schemes) of virtual excitations with regard to a set of bound reference configurations as well as on the MCDHF expansion of all atomic states involved. A first implementation of these approximate Green functions has been realized within the framework of Jac, the Jena Atomic Calculator, and will facilitate further studies on multi-photon and multiple electron (emission) processes.

While the approximate Green functions can be readily decomposed into channels (continua) of well-defined symmetry J, the detailed representation of these channels require special care. Often, the size of the associated CSF basis increase very rapidly and limits the (number of) excitations schemes that remain feasible for practical applications. Further work is necessary also to address questions about the continuous part of the spectrum, especially if two or more *free* electrons contribute to the many-electron basis. In the implementation above, these questions and the associated difficulties can be answered (distinguished) by just using different approaches (::AbstractGreenApproach). With the given classification of the Green functions, we hope to stimulate a more elaborate discussion and use of approximate atomic Green functions in the future.

## Figures and Tables

**Table 1:** Useful excitation schemes for the construction of approximate Green functions (channels).

Excitation Scheme & brief Explanation and Implementation
(De-)excitation of a single electron from a set of (non-relativistic) reference configurations: This includes all possible excitations and de-excitations of a single electron into configurations with the *same* number of electrons, but with up to *one* (free) electron in the continuum. Further restrictions can be formulated for the shells that are taken into account for excitations; cf. the data structure DeExciteSingleElectron and Table 2.
(De-)excitation of two electrons from a set of (non-relativistic) configurations: Analogue to above but by including all possible excitations and de-excitations of up to *two* electrons. These configurations then enables one to represent *N*-electron scattering states with either one *or* two free electrons within the continuum; cf. DeExciteTwoElectrons.
Add a single electron to a set of configurations: This scheme generates all possible configurations with one *additional* electron but without any further replacement of the occupied orbitals in the reference configurations. Further restrictions can be formulated for the shells that are taken into account for the excitations of the additional electron; cf. AddSingleElectronWithoutHoles.
Remove a single electron from a set of configurations: Generates all configurations with *one* electron less in any of the given subshells; cf. RemoveSingleElectron.
Remove two electrons from a given set of configurations: Analogue as above but with *two* electrons less in any (pair of) subshell; cf. RemoveTwoElectrons.
Excite one electron and capture another one: Generates all configurations with *one* electron excited with regard to the reference configurations, and with another electron captured in given high-nℓ subshells. This increases the number of electrons by one; cf. ExciteByCapture.

**Table 2:** Selected data structures of the Jac toolbox that are relevant for the generation of approximate Green functions.

Data Structure & Brief Explanation
AbstractExcitationScheme: defines an abstract and a number of concrete data types to distinguish between different schemes for generating configuration lists as they frequently occur in Green function and cascade computations; see Table 1 for details.
AbstractGreenApproach: defines an abstract and a number of concrete data types for approximating a many-electron Green function by diagonalizing the Hamiltonian matrix in a CSF basis with well-defined total symmetry J. This abstract type presently comprises the concrete data types SingleCSFwithoutCI, CoreSpaceCI, and DampedSpaceCI; see text for further explanations.
AbstractRepresentationType: defines an abstract data type to work with and discriminate between different atomic representations in Jac. A present, valid concrete types are MeanFieldBasis to generate a set of mean-field orbitals as well as an associated mean-field basis; CiExpansion to generate a configuration-mixed representation of all levels from a given set of reference configurations; RasExpansion to generate a restricted active-space expansion; and GreenFunction to generate an approximate (many-electron) Green functions. Further representations (concrete types) of this abstract data type might be added in the future.
GreenChannel: defines a data structure for a single symmetry channel of an approximate Green (function) expansion; cf. Figure 3.
GreenExpansion: defines a data structure to generate an approximate Green (function) expansion as associated with a given set of reference configurations, excitation scheme and a selected approach for the representation of the (pseudo-) ASF; see also the middle panel of Figure 2 and the example in Section 3.3.
GreenSettings: specifies additional parameters that help control the computation and generation of an approximate Green (function) expansion; cf. lower panel in Figure 2.

**Table 3:** Excitation energies $\;E_{\nu }\;-\;E_{o}\;$ of the seven low-lying levels from the Green function channel $\;J=1^{-}\;$. Energies are shown relative to the $\;1s^{2}2s^{2}2p^{6}\;{\;}^{1}S_{0}\;$ ground level and are compared with data available from the NIST Atomic Spectra Database [59]. Results are shown for three computational models. The use of a (I) diagonal CSF basis with $n^{(}\max)=15$ and ℓ=[0,1,2]; (II) the same but for the full Dirac-Coulomb Hamiltonian; and (III) the Dirac-Coulomb Hamiltonian but by including additional (double) excitations with regard to the $\;1s^{2}2s^{2}2p^{6}\;$ reference configuration. See text for further explanations.

		Excitation Energies [eV]			
Level		I	II	III	NIST [59]
$\;2s^{2}2p^{5}3s\;$	${}^{3}P_{1}$	254.21	253.26	252.66	252.0784
	${}^{1}P_{1}$	256.67	255.81	255.23	254.3889
$\;2s^{2}2p^{5}3d\;$	${}^{3}P_{1}$	293.60	292.49	291.80	291.5371
	${}^{3}D_{1}$	297.36	296.43	295.85	295.2012
	${}^{1}P_{1}$	301.48	300.88	300.17	298.9385
$\;2s^{2}2p^{5}4s\;$	${}^{1}P_{1}$	337.68	337.23	336.66	335.280
	${}^{3}P_{1}$	340.64	339.43	338.87	337.361

**Table 4:** Selected (second-order) properties and atomic processes that require a summation over one or several many-electron continua, i.e., a simple access to a proper representation of Green function channels.

Property or Process & Brief Explanation
**Frequency-dependent (ac) polarizability:** The polarizability of an atom in level (αJ) generally describes its response to the radiation field in second-order perturbation theory, that is, due to the absorption and re-emission of (laser) photons. For a sufficiently weak time-harmonic (ac) electric field, one often distinguishes between the scalar, vector and tensor polarizabilities in open-shell atoms.
**Second-order Zeeman shift:** This shift describes the dominant frequency correction to the Zeeman splitting in time-periodic (ac) laser fields and is known to play a major role in atomic-clock studies.
**Two-photon absorption & decay:** Simultaneous absorption or emission of two photons of identical or different frequencies in order to (de-) excite an atom from an initial to some fine level. This is a non-linear optical process since the (absorption) rate is proportional to the square of the light intensity, $\;W^{\;(2)}\;\propto \;I^{2}\;$. In multi-photon absorption, more generally, the rate for an *N*-photon absorption is $\;W^{\;(N)}\;\propto \;I^{N}\;$.
**Two- and multi-color photoionization:** In this process, the absorption of two or more photons leads to the emission of one or several electrons. This process has been considered in both, the weak and strong-field regime, and it comprises the two-photon direct and sequential double ionization.
**Rayleigh-Raman scattering:** The elastic Rayleigh and inelastic Raman process can be described in second-order perturbation theory in terms of two-photon amplitudes for a transition from levels $\;|\alpha_{i}\;J_{i}\rangle \to |\alpha_{f}\;J_{f}\rangle \;$, quite analogue to the amplitude (6).
**Radiative Auger emission:** This process results in the simultaneous emission of an electron and a photon, which *share* the transition energy, and mainly occurs for inner-shell excited atoms and ions.
**Single-photon double ionization:** This ionization process refers to the simultaneous emission of two electrons due to the absorption of *one* photon: $\;\;A+\hbar \omega \to A^{2+}+e_{1}^{-}+e_{2}^{-}\;$. This double ionization has been modeled also by (single-electron) knock-out and shake-off processes, although a detailed description should be based on second-order transition amplitudes, with the inter-electronic interaction as one of the perturbations.
**Double Auger decay:** This decay process gives rise to the simultaneous emission of two electrons from an inner-shell excited atom or ion; the double Auger rate amounts for inner-shell transitions to about 10 % of the single autoionization rate. This process provides valuable information about the inter-electronic correlations.
**Radiative double-electron capture:** The simultaneous capture of two electrons in ion-atom collisions may lead to the emission of energetic photons but require to properly understand the role of inter-electronic interactions.
**Pair production and annihilation:** This second-order quantum-electrodynamical process generally requires the integration over the complete spectrum of the ion but can be estimated by means of approximate Green functions.

## Data Availability

All data are given explicitly within the text.
